# High Expression of Antiviral Proteins in Mucosa from Individuals Exhibiting Resistance to Human Immunodeficiency Virus

**DOI:** 10.1371/journal.pone.0131139

**Published:** 2015-06-19

**Authors:** Sandra Milena Gonzalez, Natalia Andrea Taborda, Manuel Gerónimo Feria, David Arcia, Wbeimar Aguilar-Jiménez, Wildeman Zapata, María Teresa Rugeles

**Affiliations:** 1 Grupo Inmunovirología, Facultad de Medicina, Universidad de Antioquia UdeA, Medellín, Colombia; 2 Grupo Infettare, Facultad de Medicina, Universidad Cooperativa de Colombia, Medellín, Colombia; Rush University, UNITED STATES

## Abstract

**Background:**

Several soluble factors have been reported to have the capacity of inhibiting HIV replication at different steps of the virus life cycle, without eliminating infected cells and through enhancement of specific cellular mechanisms. Yet, it is unclear if these antiviral factors play a role in the protection from HIV infection or in the control of viral replication. Here we evaluated two cohorts: i) one of 58 HIV-exposed seronegative individuals (HESNs) who were compared with 59 healthy controls (HCs), and ii) another of 13 HIV-controllers who were compared with 20 HIV-progressors. Peripheral blood, oral and genital mucosa and gut-associated lymphoid tissue (GALT) samples were obtained to analyze the mRNA expression of ELAFIN, APOBEC3G, SAMHD1, TRIM5α, RNase 7 and SerpinA1 using real-time PCR.

**Results:**

HESNs exhibited higher expression of all antiviral factors in peripheral blood mononuclear cells (PBMCs), oral or genital mucosa when compared with HCs. Furthermore, HIV-controllers exhibited higher levels of SerpinA1 in GALT.

**Conclusions:**

These findings suggest that the activity of these factors is compartmentalized and that these proteins have a predominant role depending on the tissue to avoid the infection, reduce the viral load and modulate the susceptibility to HIV infection.

## Background

The exposure to HIV does not always lead to infection, and among those who acquire the virus the clinical course is heterogeneous. HIV-exposed seronegative individuals (HESNs) are frequently exposed to the virus without clinical or serological evidence of infection, suggesting the existence of mechanisms that prevent infection [1]. In addition, there is a small fraction of infected individuals (5–10%) who remain symptomless for more than 10 years, maintaining a relatively high CD4+ cell count (>500 CD4+ cells/μl) without antiviral therapy; they are known as long-term non-progressors (LTNP) [2]. However, the characterization of these individuals is difficult since the follow-up time to define them as LTNP is too long. Recently, a new phenotype of seropositive individuals was described, that exhibits a spontaneous and sustained control of viral replication (viral load <2000 copies/mL) at least for one year in the absence of antiretroviral therapy, known as HIV-controllers [3]. This phenotype facilitates the characterization of resistance mechanisms to AIDS progression.

Several studies in HESNs and HIV-controllers have been aimed at determining the natural resistance mechanisms to avoid infection and AIDS progression; so far, several immune components, host genetic variants, as well as soluble factors have been associated with this protection [4]. Antiviral proteins are currently the focus of several studies as they exhibit a potent HIV inhibitory activity. Some of the most important factors include: i) ELAFIN (specific elastase inhibitor), an antimicrobial molecule that inhibits HIV in a dose-dependent manner by affecting HIV attachment and transcytosis in epithelial cells [5] [6]; ii) SerpinA1 (alpha 1-antitrypsin), a serine protease inhibitor that prevents viral entry and regulates immune responses by inhibiting proteases and reactive oxygen species [7][8], and reducing the production of proinflammatory cytokines [9]; iii) RNase 7, an antimicrobial peptide with ribonuclease activity [10]; iv) APOBEC3G (apolipoprotein B mRNA-editing enzyme catalytic polypeptide-like 3G), which impairs proviral integration into the host genome [11] [12]; v) TRIM5α (tripartite motif (TRIM) proteins) may block infection at the post-entry pre-integration phase by promoting viral capsid degradation [13], and vi) SAMHD1 (SAM domain and HD domain-containing protein 1), which blocks reverse transcription [14] and degrades viral RNA [15]. Therefore, the purpose of this study was to evaluate the transcriptional expression of soluble factors in HESNs and HIV-controllers to improve the understanding of the mechanisms involved in natural resistance to HIV infection and progression; this could lead to the identification of new therapeutic targets to control this infection.

## Materials and Methods

### Population and samples

This cross-sectional study involves two cohorts of individuals: i) HIV negative individuals: HESNs and non-exposed healthy controls (HCs), and ii) HIV infected individuals: HIV-controllers and HIV-progressors.

#### i) HIV negative cohort

Fifty-eight HESN individuals, who belong to a Colombian serodiscordant couples cohort followed since 2007 [16], were recruited from HIV comprehensive care programs in Santa Marta and Medellín, Colombia. Most of them (80%) are heterosexuals and the remaining are bisexuals. None of the individuals included in this study had any sexually transmitted disease (STD) at sampling. However, 27% of HESNs reported previous events of STDs, which could have increased their risk to acquired HIV; however, they preserved the seronegative status.

The inclusion criteria for the HESN subjects were previously reported [1,17]. The HESNs in this study had an average of 8 unprotected sexual intercourses per month, within 2 years of enrollment, with a seropositive partner (SP) with detectable viral load [16], Moreover, they were negative for the HIV ELISA test, proviral HIV DNA and other infectious diseases at sampling.

Fifty-nine HC adult volunteers with similar demographic backgrounds regarding HESNs were also included. They had a negative HIV test, fewer than 2 partners in the last 2 years and self-reported no risk behaviors for HIV infection.

Samples from oral, vaginal and endocervical mucosa for women were taken from both, HESNs and HCs as previously described [1]. Oral mucosa samples were obtained using a cytobrush. As many cells as possible were collected by rubbing the brush against the oral mucosa. The vaginal samples were taken using a cervical cytobrush that was inserted and rotated 360° in all vaginal walls. Another cytobrush was gently inserted 1cm into the endocervix and rotated 360° [1]. All samples were stored in RNAlater (QIAgen, Valencia, CA) at -70°C. Peripheral blood mononuclear cells (PBMCs) were obtained by gradient centrifugation only from 23 HESNs and 38 HCs out of the total cohort.

#### ii) HIV infected cohort

Two groups of HIV-infected individuals from Medellin, Colombia were included: One group of 13 HIV-controllers defined as previously [3]. Briefly, they had a positive HIV diagnosis at least 1 year before enrolment, were highly active antiretroviral therapy (HAART) naïve and exhibited viral loads below 2000 copies/mL. The second group included 20 HIV-progressors with viral loads between 10.000 and 100.000 copies/mL and was HAART naïve at sampling.

HIV infected individuals were mostly men who have sex with men (MSMs) (72%) and acquired the infection by sexual intercourse.

PBMCs were obtained by gradient centrifugation. In addition, biopsy from gut-associated lymphoid tissue (GALT) was obtained as previously described [18]; Briefly, rectosigmoidoscopy was performed and mucosal tissue was obtained at 10 cm from anal verge using a flexible sigmoidoscope with a single endoscopy biopsy forcep FB FB-24K-1 (Olympus America Corp, Melville, NY, USA). The biopsies were stored in RNAlater (QIAgen, Valencia, CA) at -70°C. Then, tissue was digested using collagenase type II from Clostridium histolyticum (Sigma-Aldrich, St Louis, MO, USA) diluted in RPMI-1640 and 7.5% fetal bovine serum (Gibco-BRL, Grand Island, NY, USA), and RNA was extracted directly from isolated cells.

This study was approved by the Bioethical Committee, Universidad de Antioquia; all individuals signed an informed consent prepared according to the Colombian Legislation, Resolution 008430/1993 and approved by the Ethical Committee CBE-SIU of Universidad de Antioquia (certificates 11-08-352 and 13-08-520).

### Real time RT-PCR quantification

Total RNA was extracted from PBMCs and mucosa samples from HESNs and HCs using TRizol Reagent (Invitrogen, Carlsbad, CA), following the manufacturer’s instructions. For PBMCs and rectal mucosa from HIV-controllers and HIV-progressors, the RNeasy Mini Kit (Qiagen, Inc., Valencia, CA, USA) was used. RNA was quantified using the Nanodrop 1000 spectrophotometer (Thermo Scientific), treated with DNase I (Thermo Scientific) and stored at -70°C until used.

To ensure that differences in the expression of soluble factors were not a consequence of the variability in the amount of fluid or cells obtained, all cDNAs were synthesized with 1000ng (PBMCs and GALT) or 400ng (oral and genital mucosal samples) of DNAse I-treated RNA, and the same volume of cDNA (2μL) was added to all PCR assays. cDNA was synthesized using random hexamers and the Revertaid H Minus Retrotranscriptase (Thermo Scientific), following the manufacturer’s instructions.

Quantitative real time PCR (qPCR) was performed using 15 μL final volume of 2 μL of cDNA, 1X Maxima SYBR green qPCR master mix kit (Thermo Scientific) and 260 μM of the following specific primers: ELAFIN (Fw: 5'-ACCTTCCTGACACCATGAGG-3' and Rv: 5'-GACCTTTGACTGGCTCTTGC-3'); SAMHD1 (Fw: 5'-CTCGCAACTCTTTACACCGTAGA-3' and Rv: 5'-TTTCCTCCAGCACCTGTAATCTC-3'); SerpinA1 (Fw: 5’ CCGCCATCTTCTTCCTGCCTGA 3’ and Rv: 5’ CCGGAGAGGTCAGCCCCATTG 3). APOBEC3G (Fw: 5’-TCTTTGTTGCCCGCCTCTAC-3’ and Rv: 5’-CACGAACTTGCTCCAACAGTG-3’); and TRIM5α (Fw: 5’-TTCTGTCAGGAGGACGGGAA-3’ and Rv: 5’-GCTTCTGCCTCAGCATCTC-3’); furthermore, RNase 7 (Fw: 5'-AACAGACACAGCGTAGCCC-3' and Rv: 5'-GGCAGGGGTCGCTTTGC-3) was also determined only in mucosa tissues. The expression of β-actin (Fw: 5'-CTTTGCCGATCCGCCGC-3' and Rv: 5'-ATCACGCCCTGGTGCCTGG-3'), and of Phosphoglycerate Kinase 1 (PGK-1) (Fw: 5'-GTTGACCGAATCACCGACC-3' and Rv: 5'-TCGACTCTCATAACGACCCGC-3'), were used to normalize the amount of RNA in the samples from HESNs and HCs, and the expression of β2 microglobulin (Fw: 5’-GAGTATGCCTGCCGTGTG-3’ and Rv: 5´-AATCCAAATGCGGCATCT-3’) served to normalize the amount of RNA in the samples from HIV-infected individuals. The relative expression was calculated by the ΔCt method [19]. The cycling profile in all the experiments was: 95°C for 10 min, followed by 40 cycles at 94°C for 10 sec, and annealing/extension for 40 sec at 60°C. Duplicate assays were performed (SDs were less than 0.5 cycle in all assays). The results are given as median of relative expression units to the reference genes. Samples that did not amplify a target gene in the RT-PCR were excluded from the analysis of the respective gene. The number of samples included is pointed in the figure legends.

### Statistical analysis

According to Shapiro–Wilk test results, a non-parametric test (Mann-Whitney *U*-two-tailed test) was used to compare the mRNA expression of antiviral proteins between pairs of groups in each cohort (HESNs vs. HCs, and HIV-controllers vs. HIV-progressors). A *p* value <0.05 was considered statistically significant. The statistical tests were performed using the GraphPad Prism version 6.0 (GraphPad Software, San Diego, CA, USA).

## Results

### Demographic data

We analyzed samples in both cohorts including: 58 HESNs and 59 HCs; 13 HIV-controllers and 20 HIV-progressors. The demographic characteristics of the groups are shown in **Table 1.**

**Table 1:** Demographic and clinical information.

	HIV-negative cohort		HIV-infected cohort		
	HESN(n = 58)	Healthy controls(n = 59)	HIV-controllers(n = 13)	HIV-Progressors(n = 20)	*p* value
Age in years, mean ± SD	34.9 ± 10.3	32.8 ± 9.8	31.9 ± 9.3	32.3 ± 9.5	>0.500
Gender, Male:Female	24: 34	25: 34	6: 7	16: 4	N/A
Time of diagnosis in months mean ± SD	N/A	N/A	57.6 ± 45.7	63.3 ± 59.5	0.9853
Plasma HIV-1 viral load in RNA copies/mL mean ± SD	N/A	N/A	604 ± 722.8	39749 ± 42801	<0.0001
CD4^+^T cell counts mean ± SD	ND	ND	796.4 ± 211.3	520.6 ± 192.6	0,0028

N/A: N/A: Not applicable

ND: Not determined

### High mRNA levels of ELAFIN, SAMHD1, SerpinA1 and APOBEC3G in oral mucosa from HESNs

**Fig 1:**
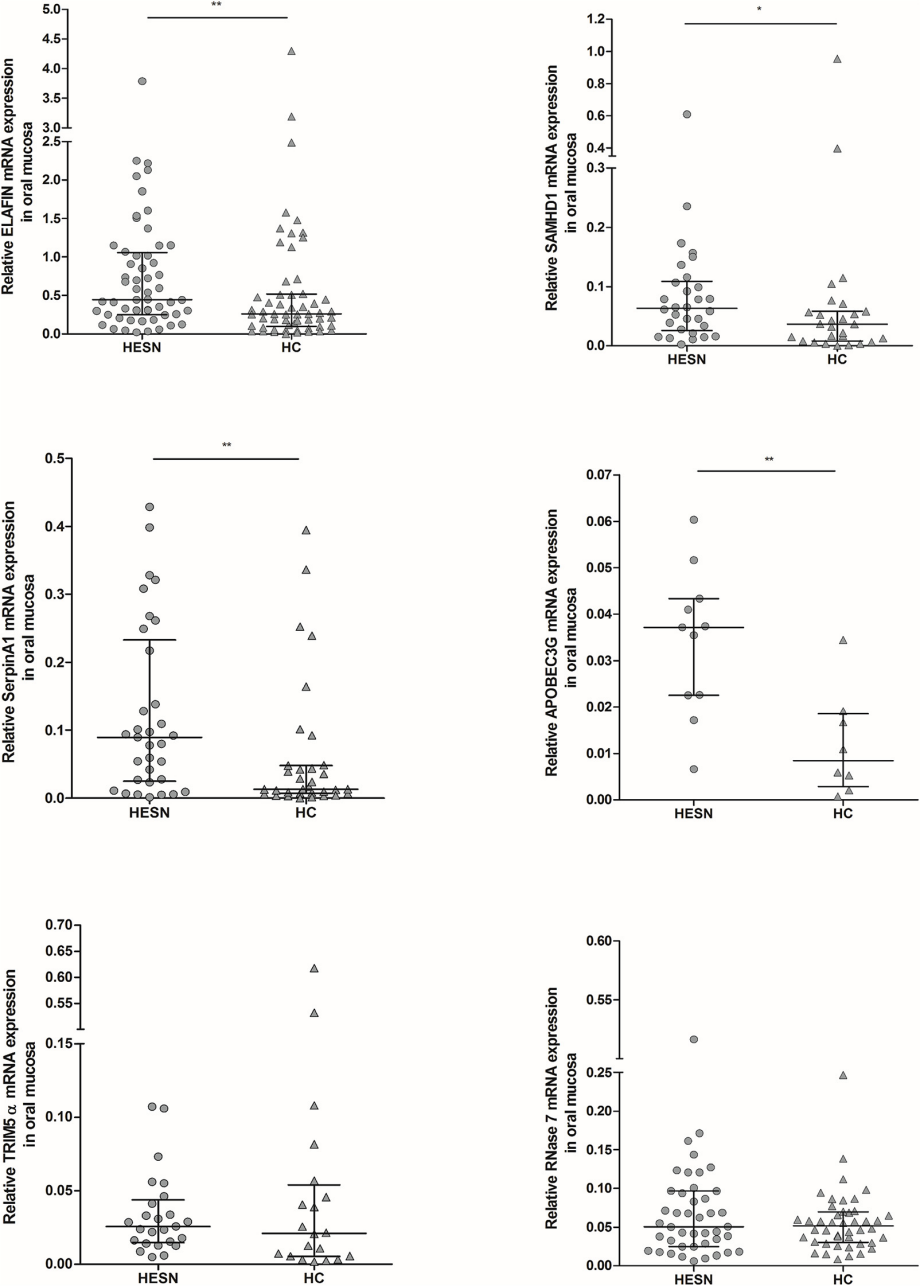
Relative mRNA expression of soluble antiviral factors in oral mucosa. RNA from oral mucosa samples of HESNs and HCs was extracted to analyze by real-time PCR the expression of ELAFIN (n HESNs = 56; n HCs = 57), SAMHD1 (n = 30; n = 27), SerpinA1 (n = 33; n = 33), APOBEC3G (n = 11; n = 8), TRIM5α (n = 25; n = 20), and RNASE-7 (n = 43; n = 44), using β-actin and Phosphoglycerate Kinase 1 (PGK-1) as reference genes to normalize the RNA content. The results are presented as median and interquartile range (25% and 75% percentiles). The statistical comparison between groups was performed using the Mann–Whitney U test with a confidence level of 95%. Significant differences are indicated at the top of the figure (*p<0.05; **p<0.01).

Higher mRNA expression of the following factors was found in HESN individuals compared to healthy individuals: ELAFIN (median 0.446 vs 0.261; p = 0.0079), SAMHD1 (0.063 vs 0.036; p = 0.0280), SerpinA1 (0.090 vs 0.013; p = 0.0071), and APOBEC3G (0.037 vs 0.008; p = 0.0034). No significant differences were observed in the expression of TRIM5α and RNase 7 in oral mucosa from HESNs and HCs (**Fig 1**).

### Increased expression of ELAFIN, SAMHD1, TRIM5α, and RNase 7 in genital mucosa from HESNs

**Fig 2:**
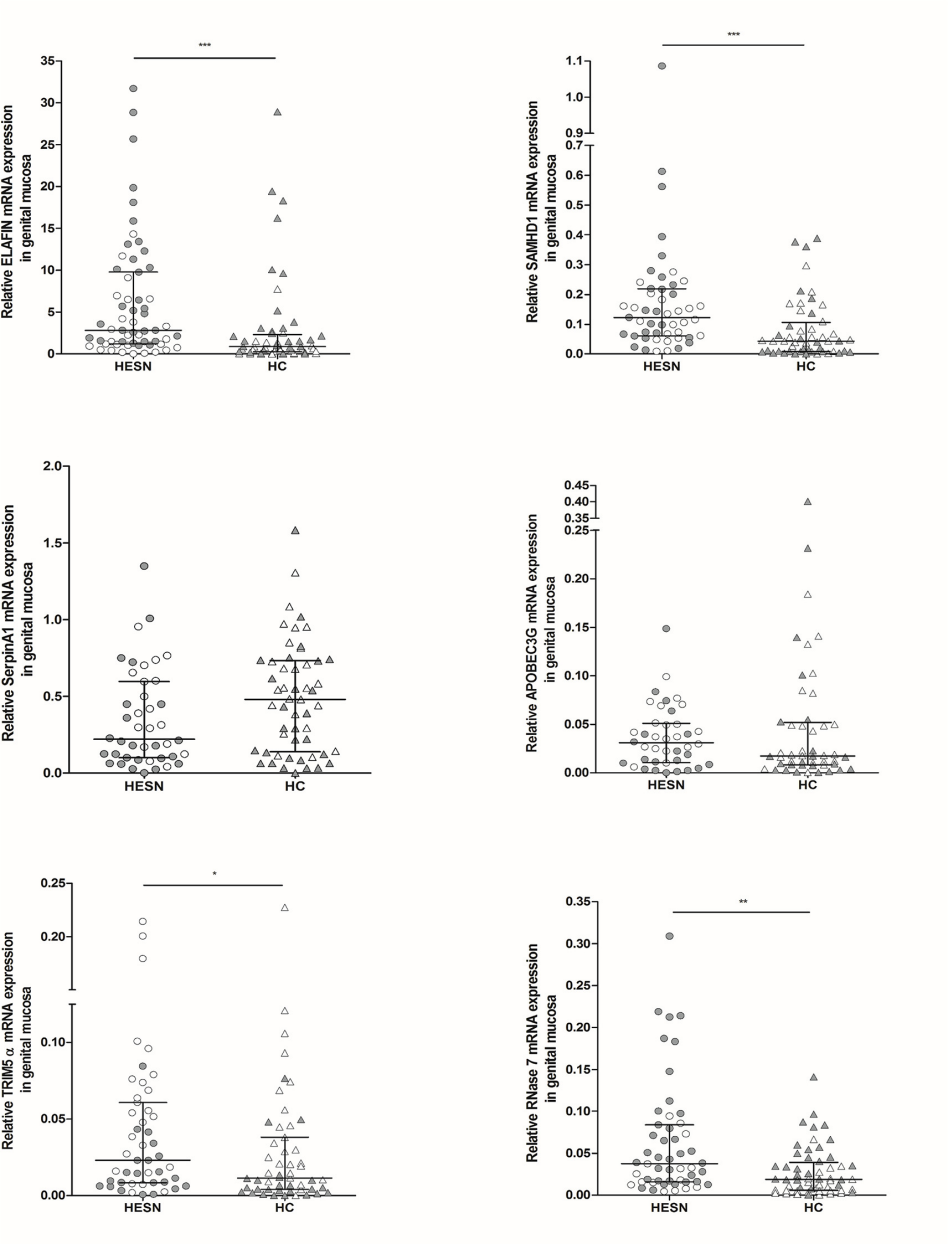
Relative mRNA expression of soluble antiviral factors in genital mucosa. RNA from both endocervical and vaginal mucosa samples of HESNs and HCs was extracted to analyze by real-time PCR the expression of ELAFIN (endocervical, n HESNs = 27; n HCs = 27); (vaginal n HESNs = 32; n HCs = 30), SAMHD1 (n = 27; n = 27); (n = 32; n = 30), SerpinA1 (n = 23; n = 25); (n = 19; n = 28), APOBEC3G (n = 20; n = 25); (n = 20; n = 23), TRIM5α (n = 25; n = 26); (n = 22; n = 25) and RNASE-7 (n = 21; n = 27); (n = 30; n = 29), using β-actin and Phosphoglycerate Kinase 1 (PGK-1) as reference genes to normalize the RNA content. White points represent to endocervical mucosa and gray points to vaginal mucosa. The results are presented as median and interquartile range (25% and 75% percentiles). The statistical comparison between groups was performed using the Mann–Whitney U test with a confidence level of 95%. Significant differences are indicated at the top of the figure (*p<0.05; ***p<0.001).

We also determined the expression level of soluble factors in both endocervical and vaginal mucosa obtained from HESNs and HCs. Since the transcriptional expression of all soluble factors was similar between these samples, we grouped the results as genital mucosa. Interestingly, the expression of ELAFIN and SAMHD1 was increased in genital mucosa from HESNs compared to HCs (2.828 vs 0.884; p<0.0001 and 0.119 vs 0.043; p<0.0001, respectively). In contrast to the findings in oral mucosa, when HESNs were compared with HCs, higher levels of TRIM5α (0.023 vs 0.012; p = 0.0406) and RNase 7 (0.037 vs 0.019; p = 0.0012) were detected in HESNs. Although mRNA expression of SerpinA1 in genital mucosa seems to be lower in HESNs, the difference was not statistically significant (0.2203 vs 0.4806; p = 0.0658). Finally, APOBEC3G was similar in both groups (**Fig 2**).

### High mRNA levels of SerpinA1 in GALT samples from HIV-controllers

**Fig 3:**
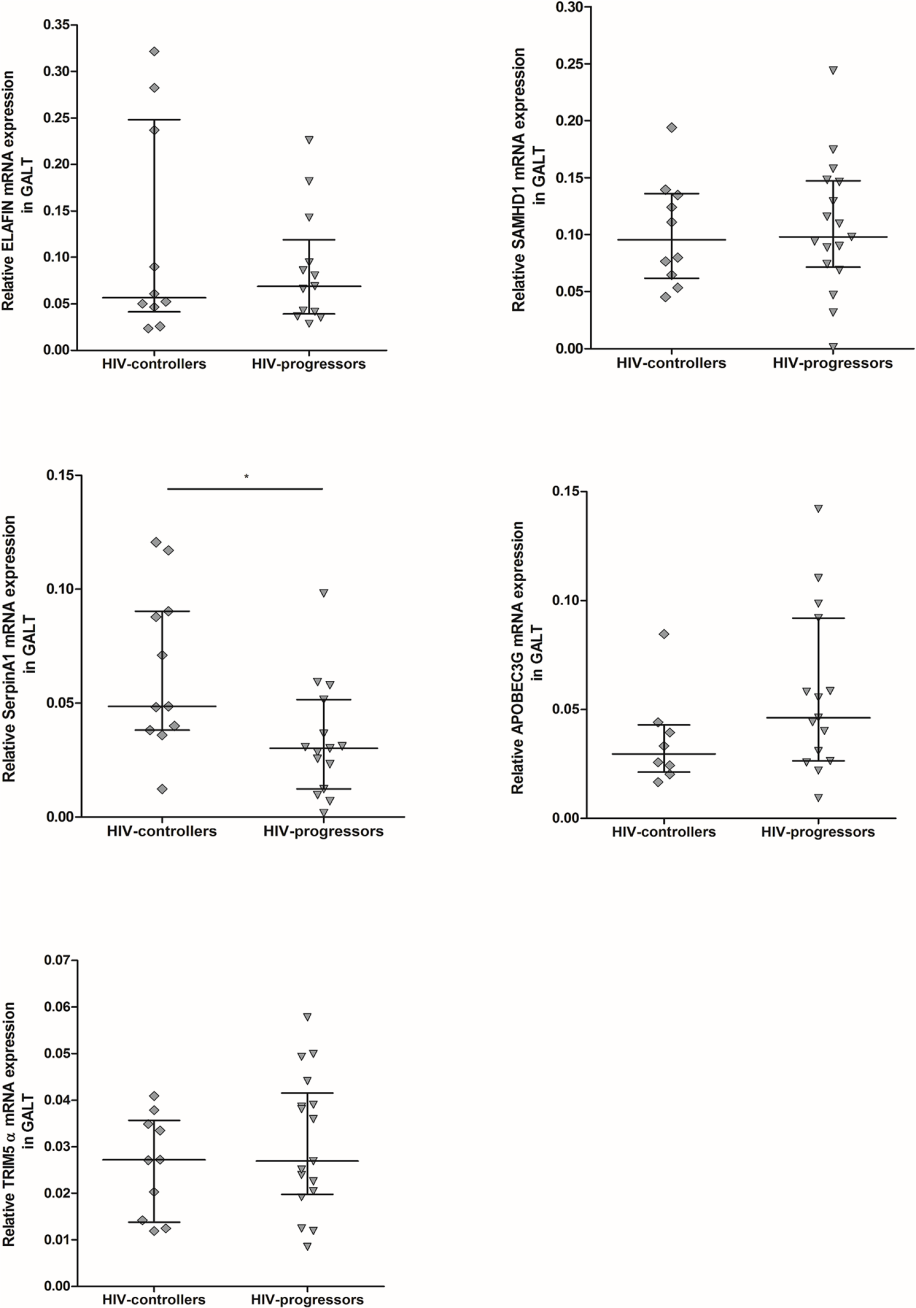
Relative mRNA expression of soluble antiviral factors in gastrointestinal mucosa. RNA from GALT samples of HIV-controllers and HIV-progressors was extracted to analyze by real-time PCR the expression of ELAFIN (n HIV-controllers = 10; n HIV-progressors = 13), SAMHD1 (n = 10; n = 17), SerpinA1 (n = 11; n = 15), APOBEC3G (n = 8; n = 15) and TRIM5α (n = 10; n = 17), using β2 microglobulin as reference gene to normalize the RNA content. The results are presented as median and interquartile range (25% and 75% percentiles). The statistical comparison between groups was performed using the Mann–Whitney U test with a confidence level of 95%. Significant differences are indicated at the top of the figure (*p<0.05).

To determine the mRNA expression level of these antiviral proteins in HIV-infected patients, we evaluated their expression in GALT as the main mucosal tissue altered during the infection. The expression of the same factors except RNase 7 was measured, since it is not expressed in GALT. Expression of SerpinA1 was higher in HIV-controllers compared to HIV-progressors (0.048 vs 0.030; p = 0.0158). The expression of the other soluble factors was similar in both infected groups (**Fig 3**). No significant correlations of SerpinA1 with either viral load or CD4+ T cells among infected individuals were observed.

### Increased expression of ELAFIN and decreased expression of SAMHD1 in PBMCs from HESNs compared to HCs

**Fig 4:**
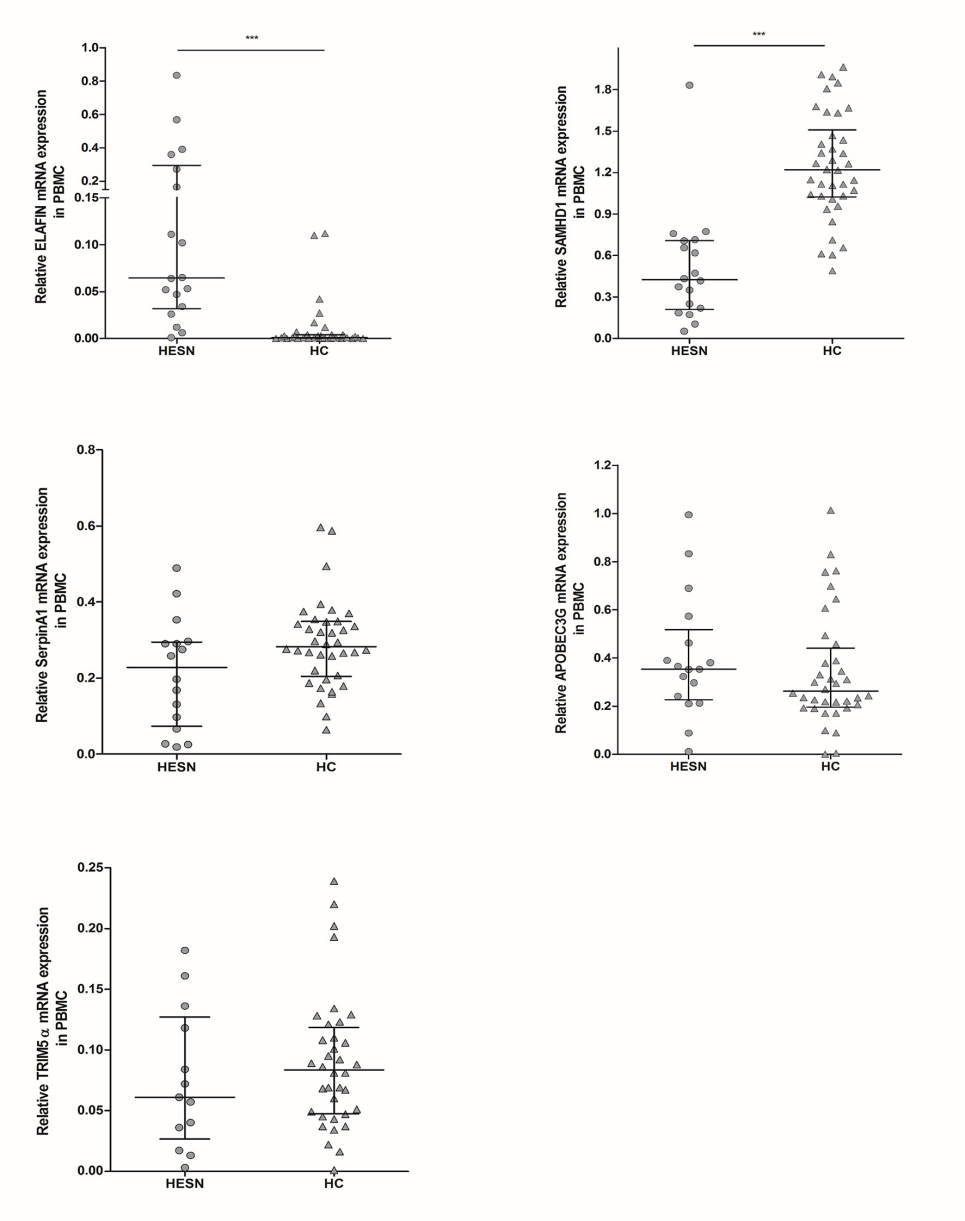
Relative mRNA expression of soluble antiviral factors in PBMCs. RNA from PBMCs of HESNs and HCs was extracted to analyze by real-time PCR the expression of ELAFIN (n HESNs = 18; n HCs = 37), SAMHD1 (n = 18; n = 38), SerpinA1 (n = 16; n = 38), APOBEC3G (n = 17; n = 36) and TRIM5α (n = 13; n = 36), using β-actin and Phosphoglycerate Kinase 1 (PGK-1) as reference genes to normalize the RNA content. The results are presented as median and interquartile range (25% and 75% percentiles). The statistical comparison between groups was performed using the Mann–Whitney U test with a confidence level of 95%. Significant differences are indicated at the top of the figure (***p<0.001).

ELAFIN levels were higher in PBMCs from HESNs compared to HCs (0.065 vs 0.001; p<0.0001); surprisingly, SAMHD1 was lower in HESNs compared to HCs (0.425 vs 1.220; p<0.0001). The expression of the other factors was similar in both groups (**Fig 4**).

In addition, when the expression of soluble factors in PBMCs from HIV-controllers and HIV-progressors were compared, no significant differences were observed (**S1 Fig**).

## Discussion

HIV infection is a complex process and exposure to the virus does not always lead to infection. This is the case of the HESNs who are a key population for identifying protective mechanisms to avoid HIV infection. There are also HIV-controllers who despite being infected, are able to control viral replication maintaining viral loads below 2000 copies/mL in the absence of antiretroviral treatment, suggesting the existence of mechanisms associated with delayed AIDS progression. Although several immune antiviral mechanisms have been previously associated with this resistance phenomenon, it is clear that they only partially explain the inhibition or control of HIV infection; consequently, other mechanisms remain to be identified.

Soluble factors constitute a remarkable mechanism to block viral replication, since they can act at different steps of the replication cycle and are becoming an interesting target for the development of novel therapeutic strategies to inhibit or reduce viral infection or replication. Here, we evaluated the transcriptional expression of the antiviral molecules ELAFIN, APOBEC3G, SAMHD1, TRIM5α, RNase 7 and SerpinA1 in PBMCs and oral and genital mucosa from HESN individuals. In addition, in HIV-controllers, the transcriptional expression of these factors in PBMCs and GALT was measured as well. In summary, in HESNs we found higher expression of ELAFIN in PBMCs and oral and genital mucosa; SAMHD1 in oral and genital mucosa; SerpinA1 and APOBEC3G, in oral mucosa; and TRIM5α and RNase 7 in genital mucosa. Furthermore, HIV-controllers exhibited higher levels of SerpinA1 in GALT. These findings suggest that the activity of these proteins is compartmentalized and that they may have a predominant role depending on the tissue. In this sense, the antiviral activity of soluble proteins achieved on the exposed mucosal tissues during sexual intercourse, could potentiate the antiviral mechanisms that avoid the infection in HESNs. Moreover, since GALT is the most important tissue for HIV replication, increased expression of antiviral proteins could reduce the viral load and modulate the impact of HIV infection on this tissue.

Previous findings suggested the role of soluble factors in the resistance to HIV infection. In this sense, ELAFIN was found over-expressed in genital samples from female HESN sex workers, compared to healthy non exposed females [20], supporting our results. Likewise, in HESNs and LTNP, increased mRNA levels of APOBEC3G in PBMCs were observed; interestingly, these levels decreased a year after termination of exposure in HESNs, suggesting that its production may be antigen driven [21]. In addition and similar to our results, higher mRNA expression of APOBEC3G in cervical tissues from HESNs compared to HCs was reported [22]. Regarding TRIM5α, it has been postulated that a single amino acid substitution increases the ability of TRIM5α to inhibit HIV replication [23]. In fact, specific alleles in individuals exhibiting resistance to HIV have been described, such as TRIM5α 136Q that is associated with higher anti-HIV activity in vitro [24].

Interestingly, the antiviral role of RNase 7 was previously reported but in the context of Dengue virus infection [25]. However, its role in genital mucosa during HIV exposure has not been described. Finally, SerpinA1 regulates immune responses by controlling inflammation, therefore reducing target cells during exposure to HIV or once the infection has been established. This protein also regulates the cytotoxic response mediated by CD8+ T cells, which is an important mechanism for controlling HIV infection [9,26]. In genital mucosa of HESNs women, higher levels of SerpinA1 compared to healthy non-exposed and HIV-infected women were reported [27]. In HIV-infected individuals, several genetic variants related with modulation of SerpinA1 expression have been described [28]. Although there are no associations of these variants with disease progression, patients with SerpinA1 deficiency have shown rapid AIDS progression [29]. To our knowledge, this is the first study suggesting the antiviral role of this protein in GALT. Finally, there are not reports indicating the association of SAMHD1 with natural resistance to HIV as suggested in this study.

Although the oral route is a less effective way to transmit HIV compared to vaginal or anal routes, it is potentially important [30]; in fact, the risk increases with the frequency of the activity, the presence of oral ulcers or sexually-transmitted infections in the oropharynx that can directly carry the virus attached to bacterial surfaces until it reaches target cells or by increasing the inflammatory milieu and the number of HIV target cells [31,32]. Some antimicrobial peptides such as human beta defensins seem to be involved in the low rate of oral HIV transmission [33]. Indeed, we previously reported that Colombian HESNs have a higher expression of defensins and SLPI in oral mucosa [1]. All these findings support the evidence regarding the antiviral role of soluble factors.

## Conclusions

Our observations suggest that soluble factors play a key role in the inhibition of HIV infection during sexual exposure and in the control of viral replication once the infection is established. They can directly block viral replication, reduce inflammation, decrease the number of susceptible target cells, therefore inhibiting infection or diminishing the associated damage induced by the virus, in case it overcomes the mucosal barriers. Investigations concerning these factors could be useful in the design of new preventive and therapeutic strategies against HIV/AIDS.

## Supporting Information

S1 Fig Relative mRNA expression of soluble antiviral factors in PBMCs from HIV-controllers and HIV-progressors. RNA from PBMCs of HIV-controllers and HIV-progressors was extracted to analyze by real-time PCR the expression of ELAFIN (n HIV-controllers = 11; n HIV-progressors = 12), SAMHD1 (n = 11; n = 15), SerpinA1 (n = 11; n = 13), APOBEC3G (n = 11; n = 14) and TRIM5α (n = 11; n = 14), using β2 microglobulin as reference gene to normalize the RNA content. The results are presented as median and interquartile range (25% and 75% percentiles). The statistical comparison between groups was performed using the Mann–Whitney U test with a confidence level of 95%. Significant differences are indicated at the top of the figure (*p<0.05).
(TIF)

## References

[1] Zapata W, Rodriguez B, Weber J, Estrada H, Quiñones-Mateu ME, Zimermman PA, et al Increased levels of human beta-defensins mRNA in sexually HIV-1 exposed but uninfected individuals. Curr HIV Res. 2008;6:531–538. 18991618 10.2174/157016208786501463 PMC4126611

[2] Piacentini L, Fenizia C, Naddeo V, Clerici M. Not just sheer luck! Immune correlates of protection against HIV-1 infection. Vaccine. 2008;26:3002–3007. 10.1016/j.vaccine.2007.11.062 18180082

[3] Walker BD. Elite Control of HIV Infection: Implications for Vaccines and Treatments. Top HIV Med. 2007;15:134–136. 17720999

[4] Zapata W, Montoya CJ, Rugeles MT. Soluble factors with inhibitory activity against type 1 Human Immunodeficiency Virus. Biomédica. 2006;26:451–466. 17176009

[5] Ghosh M, Shen Z, Fahey JV, Cu-Uvin S, Mayer K, Wira CR. Trappin-2/Elafin: a novel innate anti-human immunodeficiency virus-1 molecule of the human female reproductive tract. Immunology. 2010;129:207–219. 10.1111/j.1365-2567.2009.03165.x 19824918 PMC2814463

[6] Drannik AG, Nag K, Yao X-D, Henrick BM, Jain S, Ball TB, et al Anti-HIV-1 activity of elafin is more potent than its precursor’s, trappin-2, in genital epithelial cells. J Virol. 2012;86:4599–4610. 10.1128/JVI.06561-11 22345469 PMC3318644

[7] Pott GB, Chan ED, Dinarello CA, Shapiro L. Alpha-1-antitrypsin is an endogenous inhibitor of proinflammatory cytokine production in whole blood. J Leukoc Biol. 2009;85:886–895. 10.1189/jlb.0208145 19197072 PMC2669404

[8] Bucurenci N, Blake DR, Chidwick K, Winyard PG. Inhibition of neutrophil superoxide production by human plasma alpha 1-antitrypsin. FEBS Lett. 1992;300:21–24. 1312485 10.1016/0014-5793(92)80156-b

[9] Congote LF. Serpin A1 and CD91 as host instruments against HIV-1 infection: are extracellular antiviral peptides acting as intracellular messengers? Virus Res. 2007;125:119–134. 17258834 10.1016/j.virusres.2006.12.018

[10] Spencer JD, Schwaderer AL, Wang H, Bartz J, Kline J, Eichler T, et al Ribonuclease 7, an antimicrobial peptide upregulated during infection, contributes to microbial defense of the human urinary tract. Kidney Int. 2013;83:615–625. 10.1038/ki.2012.410 23302724 PMC3612368

[11] Mbisa JL, Bu W, Pathak VK. APOBEC3F and APOBEC3G inhibit HIV-1 DNA integration by different mechanisms. J Virol. 2010;84:5250–5259. 10.1128/JVI.02358-09 20219927 PMC2863843

[12] Kourteva Y, De Pasquale M, Allos T, McMunn C, D’Aquila RT. APOBEC3G expression and hypermutation are inversely associated with human immunodeficiency virus type 1 (HIV-1) burden in vivo. Virology. 2012;430:1–9. 10.1016/j.virol.2012.03.018 22579353 PMC3371094

[13] Stremlau M, Perron M, Lee M, Li Y, Song B, Javanbakht H, et al Specific recognition and accelerated uncoating of retroviral capsids by the TRIM5alpha restriction factor. Proc Natl Acad Sci U S A. 2006;103:5514–5519. 16540544 10.1073/pnas.0509996103 PMC1459386

[14] Baldauf H-M, Pan X, Erikson E, Schmidt S, Daddacha W, Burggraf M, et al SAMHD1 restricts HIV-1 infection in resting CD4(+) T cells. Nat Med. 2012;18:1682–1687. 10.1038/nm.2964 22972397 PMC3828732

[15] Ryoo J, Choi J, Oh C, Kim S, Seo M, Kim S-Y, et al The ribonuclease activity of SAMHD1 is required for HIV-1 restriction. Nat Med. 2014;20:936–941. 10.1038/nm.3626 25038827 PMC4318684

[16] Aguilar-Jiménez W, Zapata W, Caruz A, Rugeles MT. High Transcript Levels of Vitamin D Receptor Are Correlated with Higher mRNA Expression of Human Beta Defensins and IL-10 in Mucosa of HIV-1-Exposed Seronegative Individuals. PLoS One. 2013;8:e82717 10.1371/journal.pone.0082717 24349345 PMC3857805

[17] Young JM, Turpin JA, Musib R, Sharma OK. Outcomes of a National Institute of Allergy and Infectious Diseases Workshop on understanding HIV-exposed but seronegative individuals. AIDS Res Hum Retroviruses. 2011;27:737–743. 10.1089/AID.2010.0313 21142412 PMC3132007

[18] Rueda CM, Velilla PA, Chougnet CA, Montoya CJ, Rugeles MT. HIV-induced T-cell activation/exhaustion in rectal mucosa is controlled only partially by antiretroviral treatment. PLoS One. 2012;7:e30307 10.1371/journal.pone.0030307 22276176 PMC3261885

[19] Walker NJ. Tech.Sight. A technique whose time has come. Science. 2002;296:557–559. 11964485 10.1126/science.296.5567.557

[20] Drannik AG, Nag K, Yao X-D, Henrick BM, Ball TB, Plummer FA, et al Anti-HIV-1 activity of elafin depends on its nuclear localization and altered innate immune activation in female genital epithelial cells. PLoS One. 2012;7:e52738 10.1371/journal.pone.0052738 23300756 PMC3531372

[21] Vázquez-Pérez JA, Ormsby CE, Hernández-Juan R, Torres KJ, Reyes-Terán G. APOBEC3G mRNA expression in exposed seronegative and early stage HIV infected individuals decreases with removal of exposure and with disease progression. Retrovirology. 2009;6:23 10.1186/1742-4690-6-23 19254362 PMC2661038

[22] Biasin M, Piacentini L, Lo Caputo S, Kanari Y, Magri G, Trabattoni D, et al Apolipoprotein B mRNA-editing enzyme, catalytic polypeptide-like 3G: a possible role in the resistance to HIV of HIV-exposed seronegative individuals. J Infect Dis. 2007;195:960–964. 17330785 10.1086/511988

[23] Yap MW, Nisole S, Stoye JP. A single amino acid change in the SPRY domain of human Trim5alpha leads to HIV-1 restriction. Curr Biol. 2005;15:73–78. 15649369 10.1016/j.cub.2004.12.042

[24] Javanbakht H, An P, Gold B, Petersen DC, O’Huigin C, Nelson GW, et al Effects of human TRIM5alpha polymorphisms on antiretroviral function and susceptibility to human immunodeficiency virus infection. Virology. 2006;354:15–27. 16887163 10.1016/j.virol.2006.06.031

[25] Surasombatpattana P, Hamel R, Patramool S, Luplertlop N, Thomas F, Desprès P, et al Dengue virus replication in infected human keratinocytes leads to activation of antiviral innate immune responses. Infect Genet Evol. 2011;11:1664–1673. 10.1016/j.meegid.2011.06.009 21722754

[26] Wang Y, Yan H-J, Zhou S-Y, Wang Y-S, Qi H, Deng C-Y, et al The immunoregulation effect of alpha 1-antitrypsin prolong β-cell survival after transplantation. PLoS One. 2014;9:e94548 10.1371/journal.pone.0094548 24722487 PMC3983209

[27] Rahman S, Rabbani R, Wachihi C, Kimani J, Plummer FA, Ball TB, et al Mucosal serpin A1 and A3 levels in HIV highly exposed sero-negative women are affected by the menstrual cycle and hormonal contraceptives but are independent of epidemiological confounders. Am J Reprod Immunol. 2013;69:64–72. 10.1111/aji.12014 22971020

[28] Zhang L, Jia X, Zhang X, Cao J, Yang P, Qiu C, et al Alpha-1 antitrypsin variants in plasma from HIV-infected patients revealed by proteomic and glycoproteomic analysis. Electrophoresis. 2010;31:3437–3445. 10.1002/elps.201000153 20859951

[29] Potthoff A V, Münch J, Kirchhoff F, Brockmeyer NH. HIV infection in a patient with alpha-1 antitrypsin deficiency: a detrimental combination? AIDS. 2007;21:2115–2116. 17885308 10.1097/QAD.0b013e3282f08b97

[30] Herzberg MC, Weinberg A, Wahl SM. The oral epithelial cell and first encounters with HIV-1. Adv Dent Res. 2006;19:158–166. 16672567 10.1177/154407370601900128

[31] Campo J, Perea MA, del Romero J, Cano J, Hernando V, Bascones A. Oral transmission of HIV, reality or fiction? An update. Oral Dis. 2006;12:219–228. 16700731 10.1111/j.1601-0825.2005.01187.x

[32] Mantri CK, Chen C, Dong X, Goodwin JS, Xie H. Porphyromonas gingivalis-mediated Epithelial Cell Entry of HIV-1. J Dent Res. 2014;93:794–800. 24874702 10.1177/0022034514537647 PMC4126220

[33] Tugizov SM, Herrera R, Veluppillai P, Greenspan D, Soros V, Greene WC, et al HIV is inactivated after transepithelial migration via adult oral epithelial cells but not fetal epithelial cells. Virology. 2011;409:211–222. 10.1016/j.virol.2010.10.004 21056450 PMC3034249

