# Supplementary figures and images for: High Expression of Antiviral Proteins in Mucosa from Individuals Exhibiting Resistance to Human Immunodeficiency Virus
Source: PLoS One. 2015 Jun 19;10(6):e0131139. doi: 10.1371/journal.pone.0131139 (PMC4474690; doi:10.1371/journal.pone.0131139)

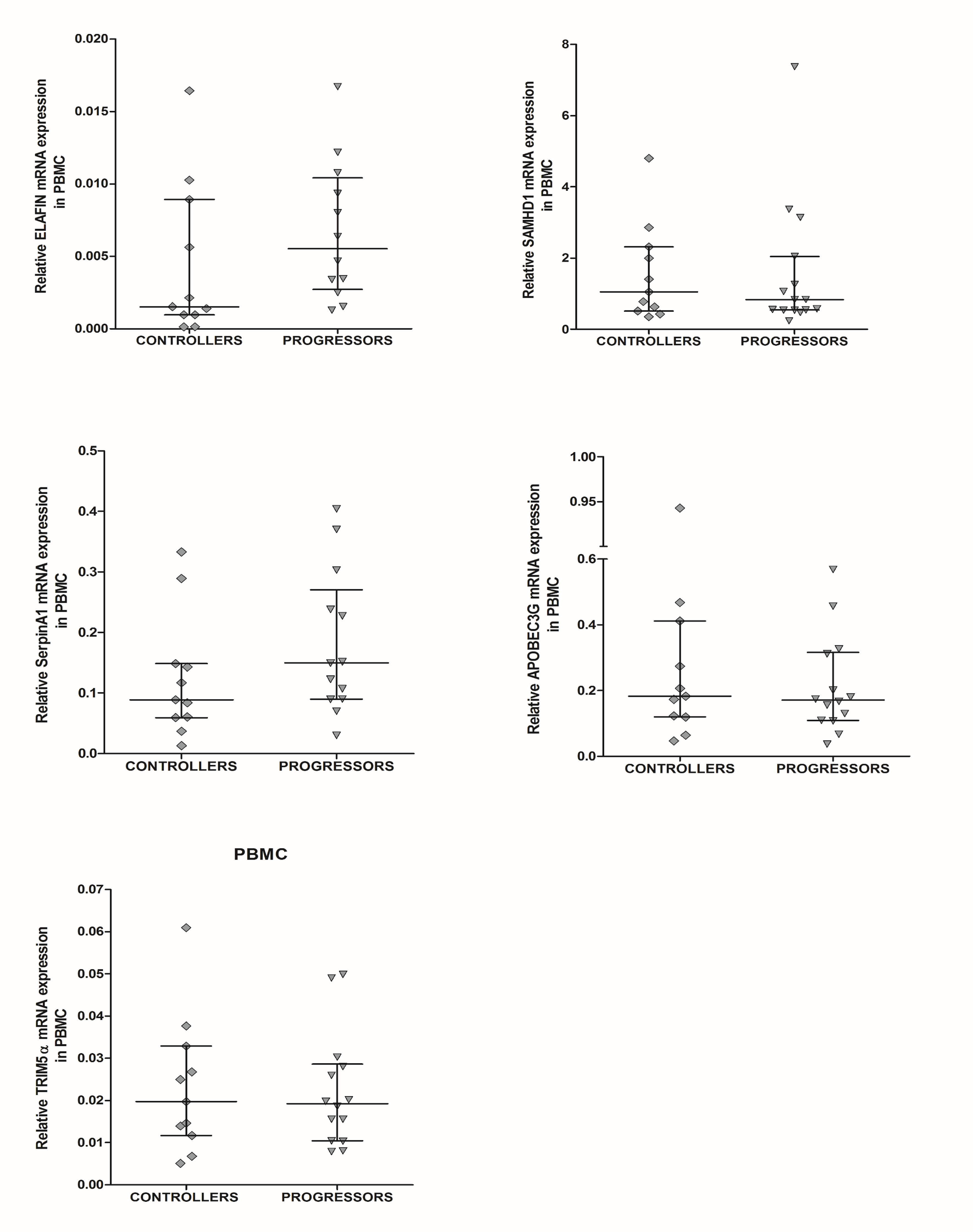

Supplement: S1 Fig — RNA from PBMCs of HIV-controllers and HIV-progressors was extracted to analyze by real-time PCR the expression of ELAFIN (n HIV-controllers = 11; n HIV-progressors = 12), SAMHD1 (n = 11; n = 15), SerpinA1 (n = 11; n = 13), APOBEC3G (n = 11; n = 14) and TRIM5α (n = 11; n = 14), using β2 microglobulin as reference gene to normalize the RNA content. The results are presented as median and interquartile range (25% and 75% percentiles). The statistical comparison between groups was performed using the Mann–Whitney U test with a confidence level of 95%. Significant differences are indicated at the top of the figure (*p<0.05). (TIF) [file pone.0131139.s001.tif]
